# MED1, a novel binding partner of BRCA1, regulates homologous recombination and R-loop processing

**DOI:** 10.1038/s41598-022-21495-8

**Published:** 2022-10-13

**Authors:** Harunori Honjoh, Michihiro Tanikawa, Osamu Wada-Hiraike, Katsutoshi Oda, Hirofumi Inaba, Asako Kukita, Yoshiko Kawata, Misako Kusakabe, Saki Tsuchimochi, Ayumi Taguchi, Yuichiro Miyamoto, Kenbun Sone, Tetsushi Tsuruga, Mayuyo Mori-Uchino, Yoko Matsumoto, Yutaka Osuga

**Affiliations:** 1 Department of Obstetrics and Gynecology, Graduate School of Medicine, The University of Tokyo, 7-3-1 Hongo, Bunkyo-ku, Tokyo, 113 8655 Japan; 2 Division of Integrative Genomics, Graduate School of Medicine, The University of Tokyo, 7-3-1 Hongo, Bunkyo-ku, Tokyo, 113 8655 Japan

**Keywords:** Homologous recombination, Oncogenes

## Abstract

Homologous recombination (HR) is a major repair pathway of DNA double-strand breaks and is closely related to carcinogenesis. HR deficiency has been established as a therapeutic target. The aim of this study was to elucidate the functions of a novel HR factor, Mediator complex subunit 1 (MED1), and its association with BRCA1. Formation of the MED1/BRCA1 complex was examined by immunoprecipitation and GST-pull down assays. The transcription cofactor role of BRCA1 was evaluated using luciferase assays. The roles of MED1 on DNA damage response and HR were analyzed by immunofluorescence and HR assays. R-loop accumulation was analyzed using immunofluorescence. R-loop-induced DNA damage was analyzed by comet assays. Immunoprecipitation and GST-pull down assays demonstrated that MED1 is a novel binding partner of BRCA1 and binds to the BRCT domain. Luciferase assays showed that MED1 potentiated the transcription ability of BRCT by two-fold. In MED1-depleted cells, recruitment of HR genes, such as RPA and γH2AX, to DNA damage sites was severely impaired. HR assays showed that MED1 knockdown significantly decreased HR activity. R-loop nuclear accumulation and R-loop-induced comet tails were observed in MED1-depleted cells. We conclude that the transcription factor MED1 contributes to the regulation of the HR pathway and R-loop processing.

## Introduction

The mechanisms by which genes are transcribed and transcription is dysregulated during carcinogenetic steps have been intensively researched for decades. Initiation, synthesis, and transcription of mRNA is catalyzed by the multi-subunit enzyme RNA polymerase II (Pol II) together with the basal transcription machinery, which comprises the general transcription factors and the Mediator complex^1–4^. The human Mediator complex has a molecular weight of approximately 2 MDa, with approximately 30 subunits and three modules (head, middle, and tail) and separable kinase modules. It is capable of dynamically changing its conformation to interact with various transcription regulators. During mRNA processing, the Mediator complex forms a bridge between general transcription factors on the enhancer and Pol II, leading to the formation of pre-initiator complexes (PICs), chromosomal loop formation, and transcriptional elongation of mRNA. It also exhibits several functions, including splicing and chromatin reconstitution, and regulates gene expression. Inactivation and hyperactivation of Mediator subunits is associated with the development of various malignancies^5–7^.

Carcinogenesis is a multi-step process involving the alteration of various signaling pathways. The altered gene expression patterns in cancer cells are attributed to gene mutations encoding cellular signaling molecules. Large scale genome rearrangement like fusion genes or sequence-specific variants in DNA binding motifs for protein modulators are also associated with carcinogenesis. To date, large scale cancer genome sequencing studies have revealed that gene alterations also arise in RNA processing machinery components including the Mediator complex. However, the role of these gene alterations and their contribution to carcinogenetic steps remains unclear. The first recognized carcinogenetic process with Mediator complex was the association between MED1 and breast cancer. Initially, in breast cancer tissues and cell lines, MED1 amplification and overexpression were observed^8,9^. MED1 overexpression was observed in 40–60% of breast cancers and positively correlated with the human epidermal growth factor receptor 2 (HER2) status^10,11^. In breast cancer cells, MED1 is phosphorylated in HER2-dependent manner and activates the estrogen receptor target genes^12^. HER2 activation is a major mechanism of breast cancer endocrine resistance. MED1 simultaneously blocks the estrogen receptor and the HER2 pathway, and thus MED1 is suggested to play a key role in HER2-mediated tamoxifen resistance and MED1’s potential role as a therapeutic target is expected^13–15^. Previous large scale next-generation sequence studies have also unveiled the high mutation frequencies of Mediator complex components. MED1 mutations and deep deletions are observed in approximately 5% of both uterine endometrial cancer and cervical squamous cell carcinoma. These data also suggest that the loss of function of the Mediator complex can drive carcinogenesis.

Genomic instability, manifested as alterations in chromosome number and structure and in DNA structure, such as nucleotide substitutions, insertions, and deletions, is characteristic of most solid tumors. To maintain genomic stability and counteract DNA damage, cells have DNA damage response (DDR) pathway. Recently, a novel layer of complexity in the cellular response to DNA damage has emerged with the involvement of RNA metabolism. RNA-binding proteins (RBPs) involved in different steps of mRNA, transcription, splicing, and translation can affect genome stability. For example, proteomic analysis to identify human and mouse proteins phosphorylated in response to DNA damage in ATM (ataxia telangiectasia mutated) and ATR (ATM-Rad3 related) consensus sites, revealed approximately 700 targets^16^, which included a large number of proteins involved in RNA metabolism. RBMX, an hnRNP associated with spliceosomes that influences alternative splicing, regulates HR in a positive manner, accumulates at sites of DNA damage in a PARP1-dependent manner, and promotes resistance to several DNA damaging agents^17^. DNA damage-induced changes in the phospho-proteome, acetylome, and proteome of human osteosarcoma cells treated with etoposide also include a significant number of proteins involved in RNA metabolism^18^.

The mechanism by which mRNA processing factors, including transcription and splicing factors, play a role in genome stability was first reported by Manley et al.^19^. Accordingly, mutations in mRNA processing genes leads to defects in the packaging of nascent mRNAs and, as a result, nascent pre-mRNA hybridizes with the transcribed strand, generating an RNA–DNA duplex known as the R-loop. R-loop, a three-stranded nucleic acid structure, causes genomic instability^20–23^. The R-loop impairs replication fork progression, and stalled replication machinery leads to double-strand breaks. The other DNA strand, which does not bind to transcribed RNA, is exposed in a single-strand loop and can be an additional source of genome instability. The R-loop is involved in cancer and neurodegenerative diseases, and many DNA damage repair proteins, RNA binding proteins, and non-coding RNAs participate in the processing of this transcription byproduct. Interestingly, BRCA1 and BRCA2, master regulators of DDR, are also involved in R-loop processing. BRCA1 forms a complex with senataxin and processes aberrant R-loop accumulation at transcriptional pause sites and protects the genome from endogenous DNA damage^24^. BRCA2 also prevents R-loop accumulation through its interaction with the TREX-2 mRNA export factor PCID2^25^.

Pathways related to mRNA biogenesis are unveiled to play an important role in genome stability and are an emerging hallmark of carcinogenesis. Subunits of the Mediator complex are found among candidate genes for homologous recombination pathways^26,27^. Compromised Mediator complex function may affect the generation of cancer-driving transcripts and induce aberrant transcription of critical DDR effectors, indirectly altering the cellular response to DNA damage. Although the R-loop may lead to the collapse of replication forks and generation of DSBs, which in turn leads to genome instability in cells with dysregulated mRNA processing, there is still little evidence of the participation of the Mediator complex in the DDR and genome stability. Therefore, in this study, we focused on MED1 to further elucidate the role of the Mediator complex on genome instability and cancer.

## Results

### MED1 forms a complex with BRCA1 and promotes BRCT domain transcriptional activity

**Figure 1:**
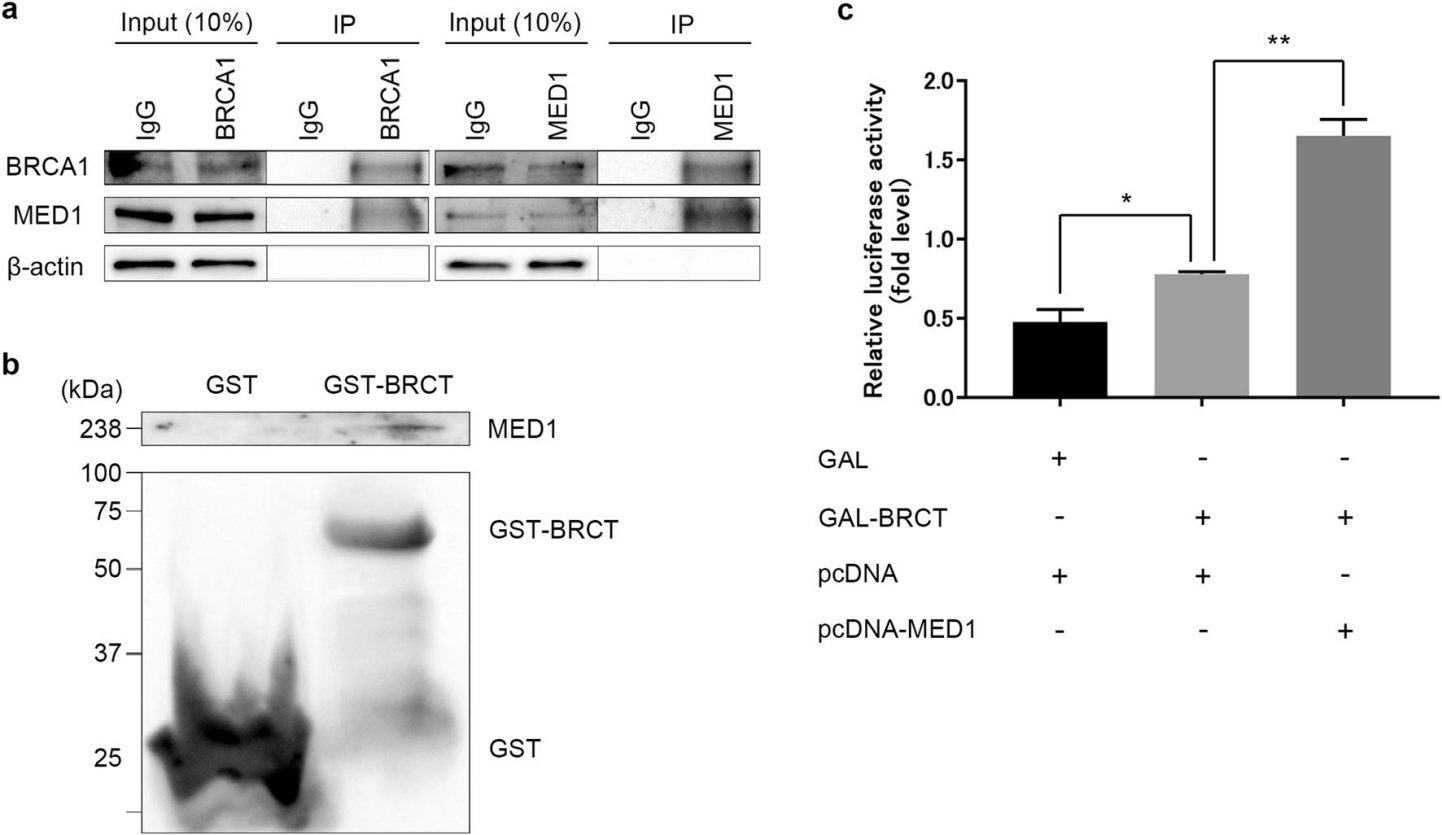
MED1/BRCA1 complex formation and evaluation of BRCT domain transcriptional activity. (**a**) Immunoprecipitation (IP) of U2OS cells. Cells were collected, washed with PBS, and dissolved in RIPA buffer to prepare whole-cell extracts. Antibodies (control IgG, anti-BRCA1, anti-MED1) were added to the supernatant and incubated, and Protein G Sepharose Fast Flow beads were added to the supernatant to purify the immune complex. Proteins bound to the beads were extracted, run on 10% SDS-PAGE, and subjected to western blotting, which confirmed the formation of a complex between MED1 and BRCA1. (**b**) GST-pulldown assay using U2OS cells. The pGEX 4T-1 or pGEX 4T-1 BRCT vectors were transformed into *Escherichia coli* and cultured. IPTG was added to the culture medium to induce protein expression, *E. coli* was harvested, and proteins were extracted. The binding of MED1 to the BRCT region was confirmed by western blotting and protein affinity columns. (**c**) Luciferase assay using 293T cells. 293T cells were seeded into 12-well dishes and transfected with each plasmid for 24 h. Firefly luciferase activity was measured simultaneously using the Dual Luciferase Reporter System (Promega, Madison, WI, USA). Renilla luciferase activity was also measured for normalization. MED1 enhanced the transcriptional activity of GAL4-BRCT by approximately 2.4-fold. Unpaired *t* test, *p < 0.05, **p < 0.01.

Immunoprecipitation (IP) of endogenous MED1 and BRCA1 was performed to determine whether MED1 interacts with BRCA1 in cells. The presence of MED1 and BRCA1 was confirmed by anti-MED1 and anti-BRCA1 antibodies in samples treated with anti-BRCA1 and anti-BRCA1 antibodies, respectively, indicating that MED1 and BRCA1 form a specific complex in human cells (Fig. 1a). To assess whether the BRCA1–MED1 association is mediated by protein phosphorylation and the presence of genomic DNA and RNA, we performed the same IP experiment in the presence of lambda-protein phosphatase, DNase, and RNase. Immunocomplex formation was not observed in these conditions, and we found that the BRCA1–MED1 association was dependent on protein phosphorylation and the existence of genomic DNA and RNA in the immunocomplex (Supplementary Fig. S1c).

To determine whether MED1 binds to the BRCT region of BRCA1, whole-cell lysates of U2OS cells were incubated with GST-BRCT (wild type) protein or GST protein, and the presence of MED1 was detected by western blotting. As shown in Fig. 1b, MED1 bound to the BRCT region.

Therefore, we examined whether MED1 overexpression affects BRCT transcriptional activity using a luciferase assay. The results show that MED1 enhanced the transcriptional activity of GAL4-BRCT by approximately 2.4-fold (Fig. 1c).

Furthermore, the effects of MED1 on cell cycle regulation were examined by flow cytometry in combination with cell cycle entrainment by double thymidine block (DTB). On the day after U2OS cells were seeded, the cells were knocked down by siRNA followed by DTB and synchronized to the G1 phase. The samples were collected at 0, 3, 6, 9, 12, and 24 h after tuning and washing thoroughly with PBS and analyzed by FACS. Twelve hours after the end of DTB, the percentage of G2/M cells was significantly increased in MED1-knockdown cells compared to that in the control (Supplementary Fig. S2a).

Transcription of p21 and GADD45α, downstream factors of the G2/M phase checkpoint pathway, are regulated by BRCA1. We examined the expression level of p21 and GADD45α by WB. U2OS cells were seeded the day before MED1 knockdown by siRNA and treated with 2 mM HU for 24 h. The results show that both p21 and GADD45α were suppressed by MED1 knockdown (Supplementary Fig. S2b), suggesting that the interaction of BRCA1 and MED1 is relevant to G2/M cell cycle regulation. A pulse-chase BrdU labelling assay showed a decreased population of early S phase cells in MED1 deficient cells in the time course of 6 h and 9 h after the BrdU labeling. This result is indicative of a delayed incorporation of BrdU in MED1 deficient cells, demonstrating that replication stress is induced by MED1 knockdown.

### MED1 mediates the transcriptional regulation and phosphorylation of ATM, Chk2, and H2AX during DSB repair

**Figure 2:**
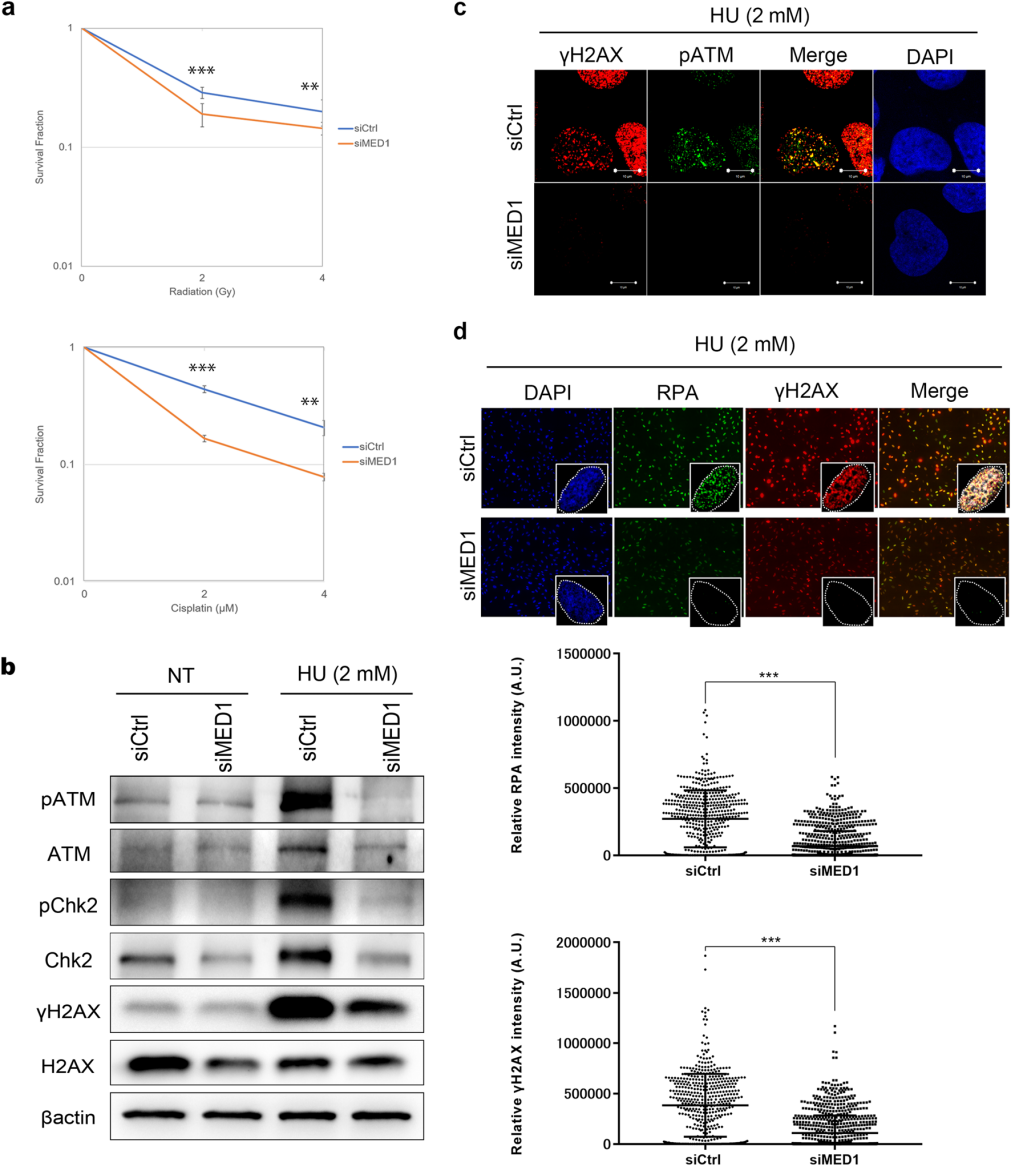
Effects of MED1 on the DNA damage response after DSB. (**a**) Colony formation assay. HeLa cells were seeded into 6 well-dishes and transfected with siRNA. After 24 h, cells were collected, reseeded, and treated with IR 0–2–4 Gy or 0–2–4 µM cisplatin. After 14 d of incubation, cells were incubated with PBS. Cells were washed, fixed in 4% paraformaldehyde, and stained with Giemsa. The number of colonies was counted, and the mean value of the three dishes was calculated to determine the survival rate against the non-treatment group. MED1-deficient cells were highly sensitive to radiation and cisplatin. (**b**) Western blotting using U2OS cells. U2OS cells were seeded in 6-well dishes and transfected with siRNAs. After incubation with 2 mM HU for 24 h from the next day, cells were collected and the protein was purified from pre-cell extracts. In MED1-depleted cells, pATM, pChk2, and pH2AX expression was suppressed during HU-induced DSB. (**c**,**d**) Immunofluorescence. U2OS cells were cultured, fixed in PBS, and pre-extracted with CSK buffer during RPA staining. After cell fixation, cells were permeabilized with 0.3% Triton X-100-containing PBS and blocked with 3% bovine serum albumin-containing PBS. Treatment was performed with primary and secondary antibodies as described above, and DAPI was used for nuclear staining. Localization was confirmed by confocal microscopy. Mobilization of pATM, γH2AX, and RPA to the DSB region was suppressed in MED1-deficient cells. Unpaired *t* test, ***p < 0.0001. *siCtrl* MISSION siRNA Universal Negative Control (Sigma Aldrich), *HU* hydroxyurea.

We further explored the role of MED1 in the DNA damage response, as BRCA1 is master regulator of homologous recombination pathway, which provides high-fidelity, template-dependent repair of tolerance of complex DNA damages including DNA double-stranded breaks (DSBs) and DNA interstrand crosslinks (ICLs). We first performed a colony formation assay in U2OS cells, which were reseeded after MED1 knockdown by siRNA and then irradiated (IR 0–4 Gy) or treated with cisplatin (0–4 μM) to assess colony formation capacity. Our results show that colony-forming ability was significantly suppressed after exposure to IR and CDDP (Fig. 2a). This suggests that the pathways of DNA damage response may be disrupted in these cells. Next, we examined the contribution of MED1 to DNA damage repair after generation of DSBs using hydroxyurea (HU), a reagent that inhibits deoxyribonucleotide synthesis. The results show that, after MED1 knockdown, the total levels of ATM, Chk2, and H2AX were reduced, as were the levels of pATM, pChk2, and γH2AX after DSB induction by HU (Fig. 2b). As for ATM, only the protein expression level under HU was reduced by MED1 knockdown.

Subsequent immunostaining under similar conditions was performed to confirm the effects of MED1 knockdown on DDR factors. Mobilization of pATM and γH2AX to the DNA damage site was inhibited under MED1 knockdown conditions during DSB induction by HU (Fig. 2c). Nuclear accumulation of γH2AX and RPA, which act downstream of ATM in the HR pathway, was also quantitatively assessed by immunostaining, and the accumulation of RPA and γH2AX in the DSB region was significantly reduced in MED1 knockdown cells (Fig. 2d). These findings suggest that MED1 may be involved in DNA damage repair.

### MED1 mobilizes RAD51/BRCA1 to the DSB site and contributes to HR activity

**Figure 3:**
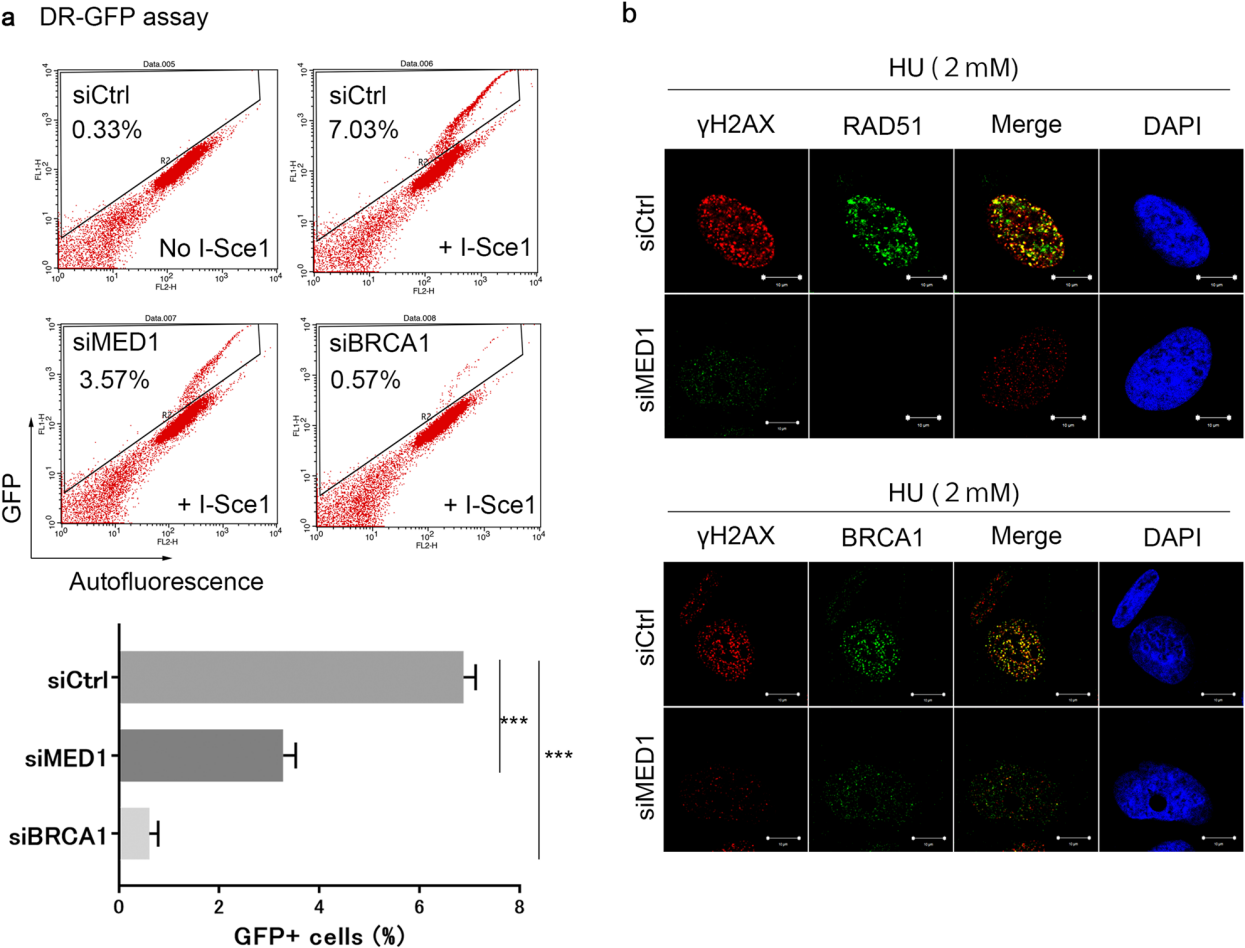
Effects of MED1 on the HR pathway. (**a**) DR-GFP assay. DR-GFP U2OS cells were seeded into 12-well plates, cultured in medium containing puromycin (1 mg/ml), and transfected with siRNA. pCMV3nls-I-SceI was transfected after 48 h of incubation. Cells were then collected, fixed in 4% paraformaldehyde, and analyzed for the number of GFP-positive cells by FACS. More than 20,000 cells per series were measured in each experiment. HR activity was reduced by approximately half in MED1-deficient cells. Unpaired *t* test, ***p < 0.0001. (**b**) Immunofluorescence in U2OS cells was performed as shown in Fig. 2c,d. Mobilization of RAD51 and BRCA1 downstream of the HR pathway was also suppressed in MED1-deficient cells. *siCtrl* MISSION siRNA Universal Negative Control (Sigma Aldrich), *HU* hydroxyurea.

We further explored the association between MED1 and homologous recombination (HR) repair using DR-GFP assays. HR activity was reduced to approximately 1/7 of the control in BRCA1 knockdown cells and to approximately 1/2 of the control in MED1 knockdown cells (Fig. 3a). We also examined the kinetics of RAD51 and BRCA1, which accumulate at DNA damage sites downstream of RPA in the HR pathway, by immunostaining, and found that, upon induction of DSBs by HU, nuclear accumulation of RAD51 and BRCA1 at DNA damage sites was clearly attenuated in MED1 knockdown cells (Fig. 3b). These findings suggest that MED1 may be involved, albeit partially, in HR activity. However, the degree of HR suppression was weaker than that of known HR factors, and HR regulation may not be the primary function of MED1.

In addition, we examined the effect of MED1 on the NHEJ pathway. When c-NHEJ activity was assessed by EJ5-GFP assay, MED1 knockdown significantly reduced GFP-positive cells compared to controls (Supplementary Fig. S3a). Furthermore, WB analysis after DSB induction by HU showed a decrease in DNA-PKcs phosphorylation during DSB in MED1 knockdown cells (Supplementary Fig. S3b). We also observed that the nuclear accumulation of 53BP1 at DNA damage sites was attenuated in MED1 knockdown cells (Supplementary Fig. S3c). This reduction of 53BP1 accumulation at DSBs may also explain the weaker suppression of HR activity in MED1 deficient cells.

### MED1 deletion causes prolonged DSB damage and delayed ATM/Chk2 phosphoprotein expression

**Figure 4:**
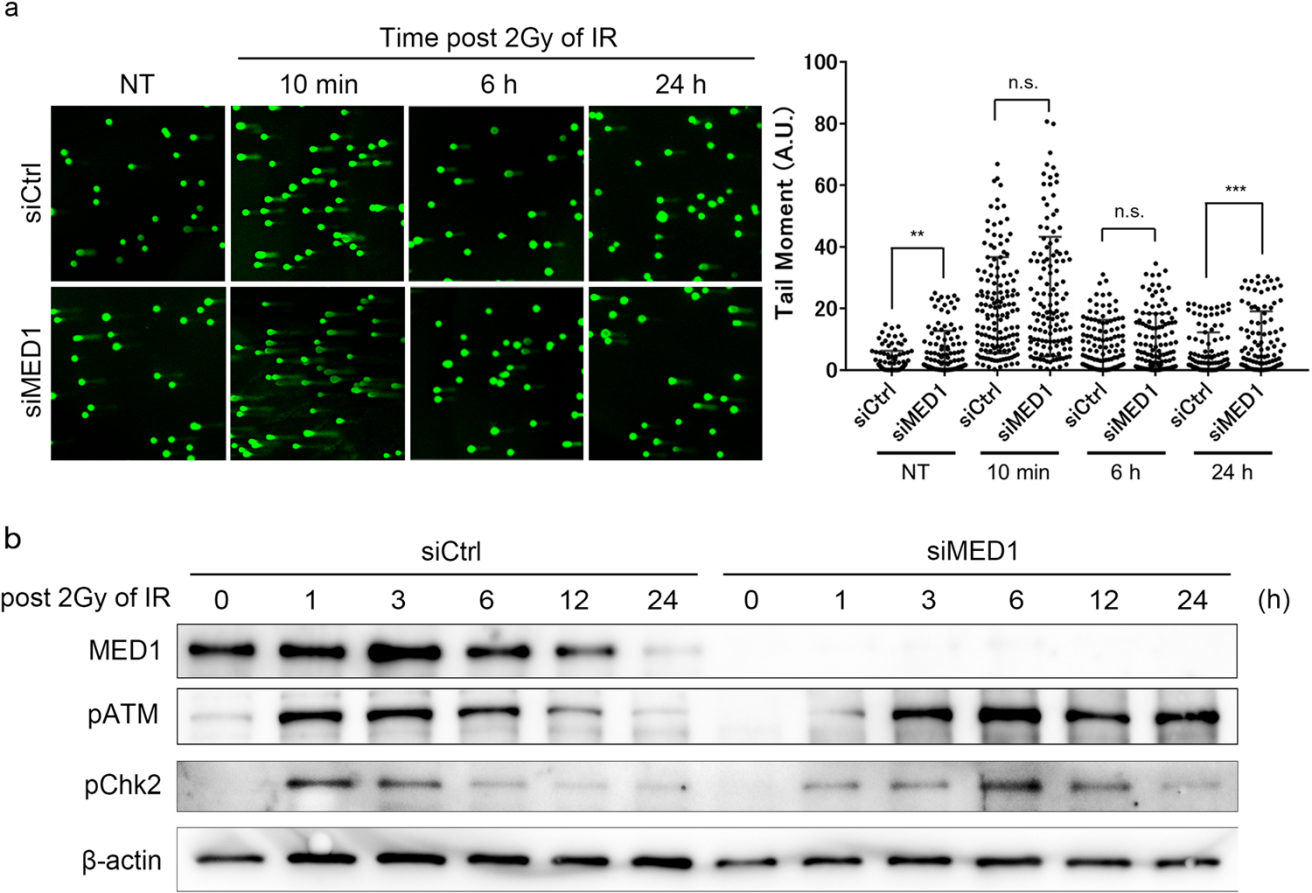
Effects of MED1 on DNA damage kinetics. (**a**) Comet assay with U2OS cells. U2OS cells were seeded onto 6-well plates and transfected with siRNA for 24 h. Cells were then irradiated with 2 Gy of IR light for 10 min. After 24 h, cells were collected using the CometAssay Kit (4250-050-K, Trevigen). Samples were processed, and nucleic acids were stained with SYBR Gold and analyzed by confocal microscopy. More than 100 comets were analyzed per sample, where the tail moment was calculated as %DNA in tail length, and DNA damage was prolonged in MED1-deficient cells 24 h after IR irradiation. Unpaired *t* test, n = 100, **p < 0.001, ***p < 0.0001. (**b**) Western blotting of U2OS cells. U2OS cells were seeded, subjected to siRNA transfection, irradiated with 2 Gy of IR, and harvested at 0–1–2–3–6–12–24 h after irradiation. Proteins were purified from whole cell extracts and analyzed by western blotting. Prolonged and delayed phosphorylation of ATM and Chk2 was observed in MED1-deficient cells. *siCtrl* MISSION siRNA Universal Negative Control (Sigma Aldrich).

We assessed whether functional deletion of MED1 affected the exacerbation of DNA damage by directly detecting DNA damage using comet assays. U2OS cells were exposed to 2 Gy IR to induce DNA damage, and cells were collected before, 10 min, 6 h, and 24 h after IR. At 10 min and 6 h after IR, no significant differences were observed in DNA damage occurring between the control and MED1 knockdown cells, whereas, at 24 h IR, DNA damage significantly increased in MED1 knockdown cells (Fig. 4a).

We then examined whether MED1 knockdown changed the expression of DNA damage response proteins over time by WB. U2OS cells were knocked down with siRNA and then irradiated with IR 2 Gy. Each cell was collected in the time course (0, 1, 3, 6, 12, and 24 h) and the protein was purified. Phosphorylation of ATM occurred after IR for 1–12 h in control cells, whereas it occurred after 3–24 h in MED1 knockdown cells. Phosphorylation of Chk2 also occurred after 1–3 h of IR in the control, whereas it occurred after 6–12 h of MED1 knockdown (Fig. 4b). These findings suggest that in MED1 depleted cells, DSB damage repair was impaired by delayed ATM/Chk2 phosphorylation, and this led to the persistent DNA damage observed in MED1 deficient cells after 24 h of IR.

### MED1 inactivation causes R-loop-induced DNA damage

Finally, we examined whether MED1 contributes to R-loop processing, a novel function of BRCA1 that has received much attention in recent years.

**Figure 5:**
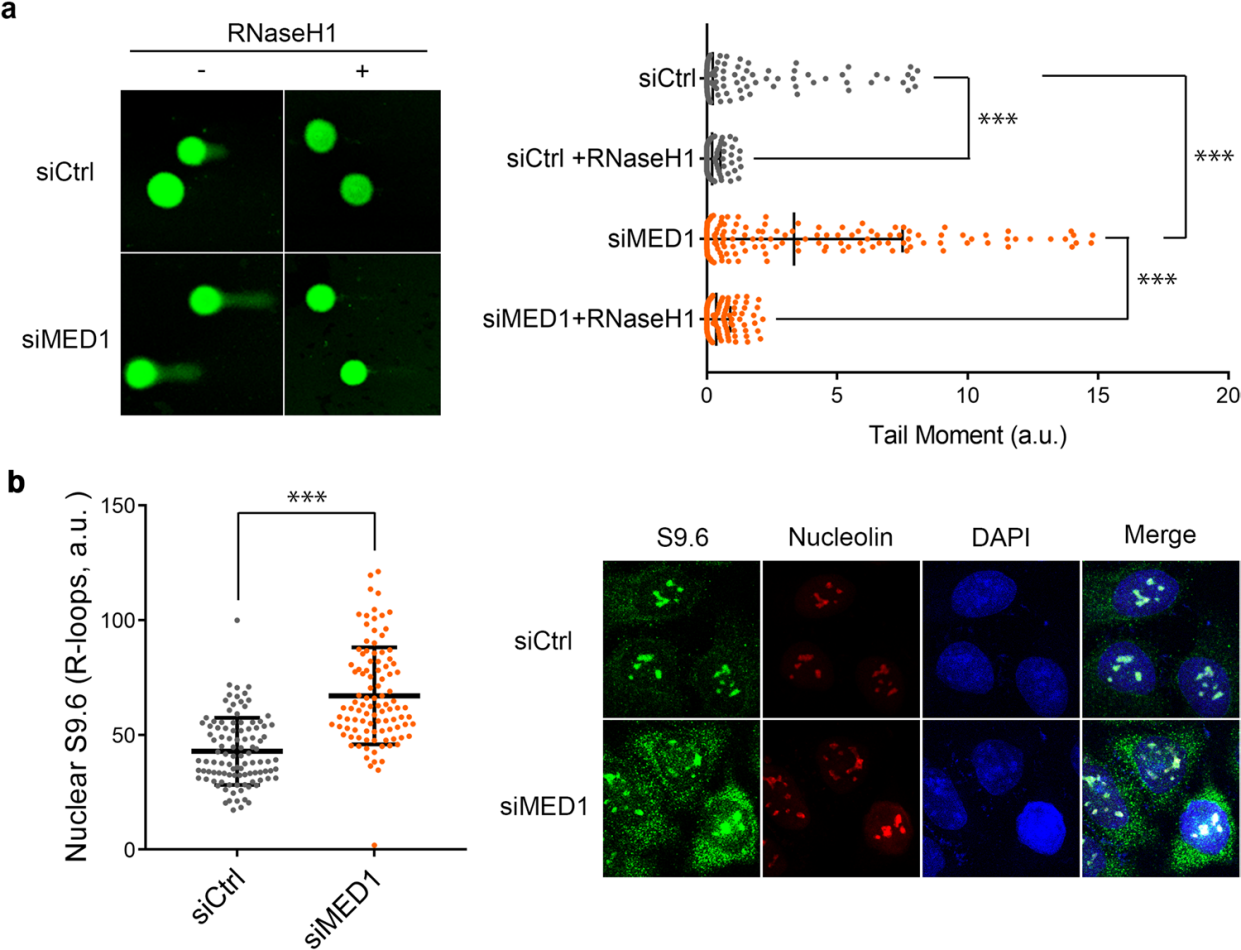
Effects of MED1 on R-loop processing. (**a**) Comet assay. U2OS cells were seeded and transfected with siRNA for 24 h. Cells were transfected with the RNaseH1 vector for 24 h. Thereafter, tail moments were analyzed. DNA damage was caused by MED1 deletion, and was prevented by forced RNaseH1 expression. Unpaired *t* test, n = 100, ***p < 0.0001. (**b**) Immunofluorescence. siRNA transfection of U2OS cells was performed the day after seeding and, after 48 h of incubation, cells were fixed with ice-cold methanol. After cell fixation, cells were permeabilized with 0.3% Triton X-100-containing PBS and blocked with 3% bovine serum albumin-containing PBS. Staining was performed using the primary and secondary antibodies described above, and DAPI was used as a nuclear stain. Localization was confirmed by confocal microscopy, and image analysis was performed using ImageJ software. After MED1 knockdown, nuclear accumulation of the R-loop increased. Unpaired *t* test, n = 100, ***p < 0.0001. *siCtrl* MISSION siRNA Universal Negative Control (Sigma Aldrich).

We performed comet assays with forced expression of RNaseH1, an R-loop degrading enzyme, in U2OS cells by siRNA MED1 knockdown, followed 24 h later by transfection of RNaseH1 vector by lipofection, and 24 h later by cell retrieval to determine whether MED1 contributed to R-loop processing. MED1 knockdown alone resulted in significantly increased DNA damage compared to the control, and the DNA damage was almost completely eliminated by the introduction of RNAaseH1 (Fig. 5a).

Subsequently, we tested whether MED1 knockdown increased nuclear R-loop by immunostaining with the S9.6 antibodies. The nucleolus was double-stained with nucleolin antibody to discriminate between the R-loop formed in the nucleus cytoplasm and the R-loop formed in the ribosomal DNA. The results show that MED1 knockdown significantly increased the nuclear R-loop luminosity (Fig. 5b).

## Discussion

This study provides evidence that MED1 interacts with BRCA1 and contributes to HR repair and R-loop processing. The evidence we uncovered here is unprecedented in suggesting that MED1 is involved in maintaining genomic stability.

Our observations suggest that MED1 forms a complex with the BRCT domain of BRCA1, the master regulator of the HR pathway, and that MED1 overexpression positively regulates the transcriptional activity of BRCA1–BRCT. The C-terminal tandem BRCT domain of BRCA1 is the hot spot region of pathogenic missense mutations in hereditary breast and ovarian cancer syndrome. Its main function is to constitute the specific phosphopeptide-binding domain of the pSer-X-X-Phe motif and to regulate HR with Abraxas/CCDC98, BACH1/BRIP1/FANCJ, CtIP, UHRF1, and others as binding partners. In addition, the BRCT region is also involved in the repair of replication fork progression arrest^28^ and R-loop processing by forming a complex with Senataxin^24^ which may be of particular importance for tumor action. As a limitation of this study, the possibility that MED1 interacts with other domains in BRCA1 cannot be ruled out, as we did not investigate the participation of the major domains in BRCA1. We also observed a loss of the MED1–BRCA1 interaction by immunoprecipitation assay in the presence of lambda-protein phosphatase, DNase, and RNase. Therefore, the interaction of MED1 and BRCA1 is dependent not only on protein phosphorylation but the existence of genomic DNA and RNA.

BRCA1 regulates the cell cycle by modulating the transcriptional activity of p21WAF1, Cyclin B1, GADD45α, and EGR1 for appropriate DNA damage repair and mitosis^29^. GADD45 binds to the cyclin B-cdc2 complex and dissociates it, preventing DNA-damaged cells from transitioning from G2 to M phase. p21 similarly prevents the transition from G2 to M phase by inhibiting cyclin-dependent kinase subsets such as cdc2. Because our data suggest that MED1 may contribute to regulation of the transcriptional activity of the BRCA1–BRCT region, we further investigated the effect of MED1 on cell cycle regulation. Our results show that MED1 regulates the expression of the BRCA1-dependent transcripts p21 and GADD45α, suggesting that MED1 may contribute to the anti-tumor cell cycle regulatory function of BRCA1.

We subsequently examined the effect of MED1 on the DNA damage response, especially on HR repair after DSB. Our results show that the colony-forming ability after exposure to IR and CDDP was significantly suppressed in MED1 deficient cells. However, compared to the BRCA1 deficient cells, the level of suppression was low (data not shown). Total protein expression of Chk2 and H2AX itself was repressed, whereas ATM was not affected by MED1 knockdown alone. However, after the induction of DSB by hydroxyurea, we observed a reduced expression of ATM in MED1 deficient cells, suggesting that MED1 is involved in the transcription of ATM under replication stress induced by hydroxyurea. When DSBs occur, ATM binds to the MRE11/RAD50/NBS1 complex and is recruited to the DSB site, where it activates and phosphorylates various substrates such as BRCA1, Chk2, NBS1, p53, and TopBP1. Thus, ATM promotes DNA damage repair and cell cycle regulation at the uppermost level and acts as a sensor of the damage response. MED1 knockdown prevented the accumulation of pATM, RPA, and Chk2 at the DSB site, suggesting that MED1 may contribute to DSB repair by directly regulating a wide range of signals downstream of ATM through the transcriptional control of ATM/CHk2/H2AX. MED1 partially contributed to HR activity, but not to BRCA1. The colony formation assay also suggests a partial role of BRCA1 via the MED1–BRCA1 interaction in the DNA damage response. BRCA1 forms diverse complexes in the HR pathway, including the BRCA1–MRN–CtIP, BRCA1–PALB2–BRCA2–RAD51, BRCA1–BACH1–TopBP1, and BRCA1–BARD1–Abraxas complexes, which play different roles in multiple stages of the HR pathway. Briefly, BRCA1 first forms a macromolecular complex that signals the presence of DNA damage and then induces DSB repair by recruiting proteins that process the excisional edges. Immunostaining showed reduced nuclear accumulation of RAD51, γH2AX, and BRCA1 at the DNA damage site after MED1 knockdown, supporting the aforementioned results. In addition, MED1 knockdown prolonged IR-induced DNA damage, and MED1 knockdown delayed and prolonged phosphorylation of ATM and Chk2. These observations suggest that MED1 regulates the HR pathway, but whether it is dependent on the transcriptional regulation of upstream factors such as CHK2 or on the BRCA1–MED1 complex, or both, cannot yet be determined. Our pulse-chase BrdU labelling assay showed the reduced DNA synthesis by MED1 knockdown and this partially explains the induced replication stress and the reduced HR activity in MED1 deficient cells.

We also examined the effects of MED1 on the activity of the NHEJ pathway, which functions as an alternative pathway to the HR pathway. Our results show reduced C-NHEJ activity by approximately 1/2 in MED1 knockdown cells and a significant suppression of DNA-PKcs phosphorylation. MED1 also affected the regulation of the upstream C-NHEJ region after DNA damage. PARP1 expression was preserved after MED1 knockdown, whereas XRCC1 transcription was repressed, suggesting that Alt-NHEJ and BRCA1-independent DNA damage repair pathways were also affected by MED1. We also observed a reduction in NHEJ activity in MED1 deficient cells by the EJ5-GFP assay, also confirmed by reduced 53BP1 accumulation at DNA damage sites induced by IR. As HR and NHEJ are balanced DNA repair pathways for DSBs, this reduced NHEJ activity in MED1 deficient cells partially explains the weaker effect on HR by MED1 knockdown compared to BRCA1. We must also consider the effects of MED1 on the transcriptional regulation and phosphorylation of ATM and H2AX. ATM and H2AX phosphorylation are upstream events of both HR and NHEJ pathways, and our observation suggests that both HR and NHEJ pathways may be repressed by reduced transcription level or phosphorylation of ATM and H2AX upon DNA damage.

Because MED1 is conjugated to the BRCT region of BRCA1, we also tested the association of MED1 with the R-loop. Our results show that DNA damage caused by MED1 knockdown was cancelled by forced RNaseH1 expression. Furthermore, MED1 knockdown induced R-loop accumulation, suggesting that MED1 may contribute to R-loop processing. Whether R-loop processing is a MED1–BRCA1 complex-dependent phenomenon and the mechanism of MED1 knockdown-induced DNA damage is open to further investigation. Components of the homologous recombination mechanism are involved in R-loop formation and genomic instability. Although the details remain unclear, the breast cancer suppressors BRCA1 and BRCA2 have been suggested to be involved in pathways that prevent R-loop accumulation^24,29,30^. Our data suggest that a novel HR factor, MED1, may be one of the factors involved in both complex HR and R-loop processing.

In this work, we showed R-loop formation by fluorescent immunostaining using S9.6 antibody, and that the induced DNA damage by MED1 depletion is rescued by overexpression of RNAseH1. We also showed by BrdU assay that replication stress may be induced under knockdown of MED1 through the mechanism of transcription–replication conflict. On the other hand, we have not been able to verify whether the results of our S9.6 antibody-based fluorescent immunostaining assay are affected specifically by RNAseH1, not by RNAse T1 or RNAse III^31^. Besides, R-loop formation in cells has recently been shown to be affected by various RNA-degrading enzymes, including RNAseH1 and RNAseH2^32^. R-loops are more robustly detected in cells using DRIP assay and RNAseq assay. However, we have not established these methods in this research, and these are the limitations of our work.

Although the present study showed that the phosphorylation signals of ATM and H2AX are reduced and both HR and NHEJ pathways, which are regulated by ATM and H2AX phosphorylation, are repressed by MED1 knockdown, the contribution of MED1 to the transcriptional regulation of DNA damage repair factors is not well defined. The decreased protein levels of H2AX at steady state and the decreased protein levels of ATM upon double-strand break induction under MED1 depletion may suggest an aspect of MED1 as a transcription factor that forms a complex with RNA polymerase II and BRCA1. It has been reported that BRCA1 forms an mRNA splicing complex with BCLAF1 to contribute the efficient transcription and splicing of DNA damage repair factors during DNA damage induction^33^, and MED1 may also be involved in this mechanism.

The reduced ATM and H2AX phosphorylation signals under MED1 knockdown may also suggest that the direct contribution of MED1-containing complexes, such as Mediator complex, to ATM phosphorylation. As for phosphorylation of ATM, the intermolecular ATM autophosphorylation and direct phosphorylation by the MRN complex have been elucidated^34,35^. On the other hand, recent studies have unveiled the mechanisms by which mRNA splicing intermediates and R-loop contribute to ATM phosphorylation^36,37^. In these studies non-dividing neuronal cells were used, indicating that replication-dependent conversion of these lesions into DSBs in S phase is not a requirement for ATM activation. The possibility that the R-loops formed by MED1 knockdown may contribute to ATM phosphorylation remains a subject for further investigation.

In conclusion, our results indicate that MED1, a novel binding partner of BRCA1–BRCT, may contribute to the maintenance of genomic stability through its contribution to the HR pathway and suppression of R-loop accumulation.

## Material and methods

### Cell culture, transfection, and treatment

U2OS osteosarcoma cells, HeLa cells, and 293T cells were purchased from ATCC and cultured in DMEM (Sigma-Aldrich, Schnelldorf, Germany) supplemented with 10% FBS (Sigma-Aldrich) and 1% antibiotics (penicillin) (Gibco; Thermo Fisher Scientific, Waltham, MA) at 37 °C and 5% CO_2_. DR-GFP U2OS cells were kindly provided by Prof. Thomas Helleday and Dr. Oliver Mortusewicz and cultured in DMEM with 10% FBS, antibiotics and 1 μg/ml puromycin (Gibco). For siRNA-mediated knockdown, cells were transfected with 5 nM predesigned Silence Select siRNAs or control siRNA (Ambion/Life Technologies) using INTERFERin (Polyplus Transfection, Illkirch, France). For plasmid transfection, jetPEI (Polyplus Transfection) was used. Chemotherapeutic treatment was performed using hydroxyurea (HU; Sigma-Aldrich) dissolved in H_2_O, and X-rays were generated using a CellRad X (Faxitron, USA).

### Small interfering RNA (siRNA)

siRNA-mediated knockdown was achieved using INTERFERin (Polyplus Transfection) following the manufacturer’s instructions. Predesigned Silence Select siRNAs or control siRNA (Ambion/Life Technologies) were used. Each siRNA was used as a pool of three target-specific siRNAs: non-targeting, Mission SIC-001 (Sigma-Aldrich); MED1, #s10889 (5′-GCUGGUCCCUUGGAUAAGAtt-3′, 3′-UCUUAUCCAAGGGACCAGCat-5′), #s10890 (5′-GACCAGUCCUUGUCUAUGAtt-3′, 3′-UCAUAGACAAGGACUGGUCtg-5′), and #s10891 (5′-CCAGUACAGGUGGUGGAUCUAAtt-3′, 3′-UUAGAUCCACCUGUACUGGta-5′); and BRCA1, #s457 (5′-GGGAUACCAUGCAACAUAAtt-3′, 3′-UUAUGUUGCAUGGUAUCCCtc-5′), #s458 (5′-CAGCUACCCUUCCAUCAUAtt-3′, 3′-UAUGAUGGAAGGGUAGCUGtt-5′), and #s459 (5′-CAUGCAACAUAACCUGAUAtt-3′, 3′-UAUCAGGUUAUGUUGCAUGgt-5′). siRNAs were transfected with INTERFERin (Polyplus Transfection) at a final concentration of 5 nM.

### Plasmid

The pCMV-I SceI plasmid and pcDNA-RNaseH1 vector were kindly provided by Prof. Thomas Helleday and Dr. Oliver Mortusewicz. The pcDNA3, pGEX 4T-1, and pRL Renilla CMV-Luc control reporter vectors were purchased from Invitrogen (Camarillo, CA), GE Healthcare (Buckinghamshire, UK), and Promega (Madison, WI), respectively. The pGEX 4T-1 BRCT, GAL4, GAL4-BRCT, and pcDNA-MED1 vectors and reporter constructs (17M8-AdMLP-luc) were described previously by Hiraike et al*.*^38^.

### Antibodies

Used primary and secondary antibodies are listed in Supplemental Table 1.

### Immunoblotting

Total cell extracts were obtained using RIPA buffer (50 mM Tris–HCl pH 8.0, 150 mM NaCl, 0.5% w/v sodium deoxycholate, 0.1% w/v sodium dodecyl sulfate, 1% w/v NP-40) with complete, EDTA-free (Roche, Basel, Switzerland), and Halt Phosphatase inhibitor cocktail (Thermo Scientific). Sample buffer (1 M Tris–HCl pH 6.8, 40% w/v glycerol, 20% w/v 2-Mercaptoethanol, 8% w/v sodium dodecyl sulfate, 0.02% w/v bromophenol blue) was added to the extracts. Equivalent amounts of lysate protein (10 μg) were used in Mini-PROTEAN TGX Precast Protein Gels (Bio-Rad) and electrophoretically transferred onto Trans-Blot Turbo Transfer Packs (Bio-Rad) using the Trans-Blot Turbo Transfer System (Bio-Rad). Proteins transferred onto membranes (Bio-Rad) were probed with appropriate primary and secondary antibodies. Target protein expression was detected using ECL Plus WB detection reagents (GE Healthcare) using Image Quant LAS 4000 mini (GE Healthcare).

### Immunoprecipitation

The formation of a MED1–BRCA1 complex in U2OS cells was analyzed by IP. Whole-cell extracts of U2OS cells using RIPA buffer were used for IP with anti-BRCA1 and anti-MED1 antibodies or preimmune IgG. The immunoprecipitate was subjected to 30 ml of protein G Sepharose 4 Fast Flow (GE Healthcare) and subsequently immunoblotted with anti-BRCA1, anti-MED1, or anti-IgG antibodies. Immunoprecipitation samples were treated with lambda protein phosphatase (Sigma Aldrich), DNase I (Sigma Aldrich), or RNase (Sigma Aldrich) according to the manufacturer’s protocol.

### Immunofluorescence

Cells plated on coverslips were fixed for 10 min at room temperature with fixative (3% paraformaldehyde [PFA], 0.1% Triton X-100, 1 × PBS). For RPA staining, pre-extraction was performed for 5 min with ice-cold 0.3% TritonX-100 in CSK buffer (100 mM NaCl, 300 mM sucrose, 3 mM MgCl_2_, 10 mM PIPES, 1 mM ethylene glycol-bis (β-aminoethyl ether)-N,N,N0,N0-tetraacetic acid). After fixation, cells were rinsed briefly with 0.05% Tween 20 in PBS twice, and then permeabilized for 10 min with 0.3% Triton X-100 in PBS. After blocking for 40 min with PBS + 3% BSA or skim milk in PBS, cells were incubated with primary antibodies diluted in PBS + 3% BSA or skim milk in PBS at 4 °C overnight in a wet chamber. Cells were again rinsed twice with 0.05% Tween 20 in PBS and then permeabilized for 10 min with 0.3% Triton X-100 in PBS. Cells were then incubated with secondary antibodies diluted in PBS + 3% BSA at room temperature in the dark for 1 h. After washing with 0.05% Tween 20 in PBS, nuclei were stained with 4′,6-diamidino-2-phenylindole (DAPI) for 5 min, washed with 0.05% Tween 20 in PBS, and permeabilized with 0.3% Triton X-100 in PBS. After rinsing with D2W, slides were mounted with ProLong Gold (Life Technologies) and visualized using a confocal microscope LSM700 (Carl Zeiss, Le Pecq, France). For foci quantification, cells were fixated in 96 well plates (BD Falcon) using the same protocol, and images were taken using BZ-X710 (Keyence, Osaka, Japan) and analyzed using Image J software (NIH, Bethesda, Maryland, USA) and BZ-X800 Analyzer (Keyence, Osaka, Japan). More than 400 cells were counted. Error bars represent SEM from three independent experiments.

### GST-pull down assay

Glutathione-S-transferase fusion proteins or GST alone were expressed in *Escherichia coli* and immobilized on glutathione Sepharose 4 B beads (GE Healthcare). GST proteins were incubated with whole-cell extracts of U2OS cells. Unbound proteins were removed, and bound proteins were eluted and analyzed by SDS-PAGE.

### Luciferase assay

293T cells were seeded (6.0 × 10^4^/well) in 12-well plates and transfected with the indicated expression vectors using jetPEI (Polyplus transfection, Ilkirch, France). Luciferase assays were performed using the dual luciferase reporter assay system (Promega). Expression vectors were co-transfected with reporter plasmid (17M8-AdMLP-Luc). As an internal transfection control, phRL CMV-*Renilla* vector (Promega) was also transfected in all experiments. After 24–48 h of transfection, cells were harvested, washed with PBS, and fixed following manufacturer’s recommendations. Luminescence was measured using an LB96V luminometer (Berthold Technologies, Bad Wildbad, Germany). Individual transfections, each consisting of triplicate wells, were repeated at least three times.

### Colony formation assay

HeLa cells (8.0 × 10^4^/well) were seeded in 6-well plates. After 24 h of incubation, endogenous MED1 was depleted with siRNA-mediated knockdown. Two days after siRNA transfection, cells were collected by trypsinization, treated with different doses of radiation, and reseeded into 6-well plates at 1 000 cells/well. Alternatively, cells were reseeded and cultured in various concentrations of cisplatin. The medium and cisplatin were replaced every 3 days. After 7 days of incubation, the cells were fixed with 100% methanol for 2 h and stained with Giemsa (Wako, Osaka, Japan). The dishes were gently washed with water and air-dried. Colonies with more than 50 cells were counted. Results are presented as the mean ± SEM from three independent experiments.

### HR and NHEJ assays

HR and NHEJ assays were performed in DR-GFP USOS and EJ5-GFP U2OS cells, respectively. Cells were seeded in 12 well plates (5.0 × 10^4^ cells/well). Twenty-four hours after transfection of siRNA against endogenous MED1 or BRCA1, or non-targeting siRNA, cells were transfected with plasmids expressing I-SceI endonuclease or an empty vector as a control. After 48 h, cells were harvested, fixed with 4% PFA, and subjected to FACS analysis to determine the efficiency of HR or C-NHEJ repair induced by I-SceI digestion, which reconstituted a functional GFP gene. GFP signals were quantitated on a FACS Navios flow cytometer (BD FACSCalibur HG, BD Bioscience, Franklin Lakes, NJ, USA) and analyzed with Cell Quest Pro software v 3.1 (BD Bioscience). For each experiment, 20,000 cells were scored per treatment group, and the frequency of recombination events was calculated from the number of GFP-positive cells divided by the number of cells analyzed. Data are presented as the mean ± SEM from three independent experiments with *p*-values determined by Student’s *t* test.

### Alkaline single-cell gel electrophoresis comet assay

U2OS cells were seeded in 6-well plates (1.0 × 10^5^ cells/well). Twenty-four hours later, cells were transfected with non-targeting siRNA or MED1 pool siRNAs using INTERFERin (Polyplus Transfection). After another 24 h, cells were treated with X-rays and incubated for 24 h before harvesting. After washing with PBS, cells were re-suspended in PBS at a concentration of ~ 1.0 × 10^6^ cells/ml. DNA damage was evaluated using the Comet Assay Kit (4250-050-K, Trevigen) following manufacturer’s instructions. Electrophoresis was run at 300 mA, 21 V for 30 min in electrophoresis buffer using the Comet Assay Electrophoresis System II (Trevigen). Slides were washed in neutralization buffer (0.4 M Tris–HCl pH 7.5) and counterstained with SYBR Gold (diluted 1:1000 in PBS) (Invitrogen). Images were acquired using a confocal microscope LSM780 (Carl Zeiss), and comet tail moment was estimated using CometScore software. At least 100 comets per sample were analyzed.

### Cell cycle analysis using double thymidine block

U2OS cells were seeded (8.0 × 10^4^ cells/well) in 6-well plates and transfected with indicated siRNAs. After 24 h of incubation, cells were reseeded in 6-well plates (2.0 × 10^5^ cells/well). For cell cycle synchronization, cells were arrested twice at the G1/S boundary using a double incubation in the presence of 2.5 mM thymidine for 15–18 h, followed by a 9-h interval of growth without the drug. Cells were released from the cell cycle blocks and harvested using trypsin at the indicated times. Cells were stained in the dark with 50 μg/ml propidium iodide (PI) (Sigma-Aldrich) at 4 °C for 30 min. Cell cycle distribution was analyzed by flow cytometry on an Epics XL instrument (Beckman Coulter, Brea, CA, USA) using Cell Quest Pro software v 3.1 (BD Bioscience, Franklin Lakes, NJ, USA). The data are presented as the mean ± SEM from three independent experiments.

### BrdU pulse chase assay

FITC BrdU Flow Kit (BD Bioscience) was used. U2OS cells were seeded (4.0 × 10^4^ cells/well) in 6-well plates and transfected with indicated siRNAs. After 48 h of culture, cells were treated with 10 μM BrdU. Cells were collected and fixed at appropriate time points (0, 3, 6, 9 h). Following the manufacturer’s protocol, cells were analyzed by flow cytometry on an Epics XL instrument (Beckman Coulter, Brea, CA, USA) using the Cell Quest Pro software v 3.1 (BD Bioscience, Franklin Lakes, NJ, USA).

### Statistical analysis

Statistical analyses were performed using GraphPad Prism 7 software (GraphPad Software, San Diego, CA). Results are shown as mean ± standard error of the mean. Data were analyzed using Student’s *t* test for paired comparisons. Statistical significance was set at *p* < 0.05.

## Supplementary Information

Supplementary Information.

## Data Availability

The datasets used or analyzed during the current study are available from the corresponding author on reasonable request.
