# Supplementary material for: MED1, a novel binding partner of BRCA1, regulates homologous recombination and R-loop processing
Source: Sci Rep. 2022 Oct 13;12:17140. doi: 10.1038/s41598-022-21495-8 (PMC9561711; doi:10.1038/s41598-022-21495-8)
Supplement: Supplementary file 1 — Supplementary Information. [file 41598_2022_21495_MOESM1_ESM.pdf]

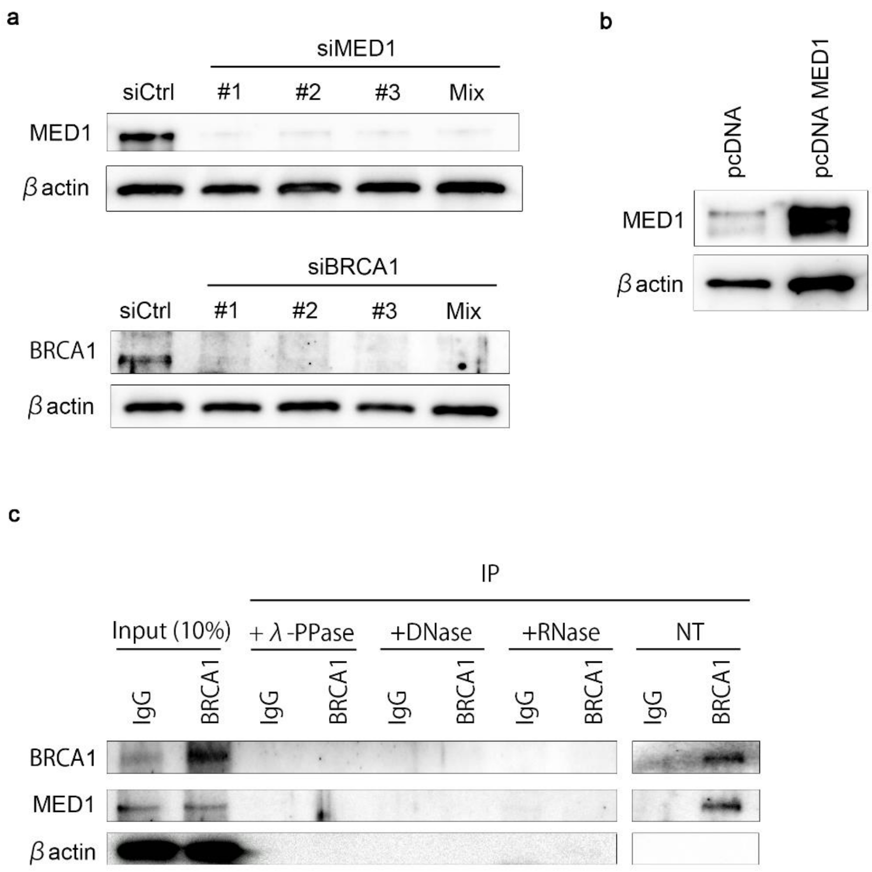

Supplementary Figure S1. Confirmation of MED1 and BRCA1 knockdown efficiency by siRNA and MED1 overexpression and additional immunoprecipitation assay.

(a) U2OS cells were seeded into 6-well plates. After 24 h of incubation, MED1 or BRCA1 was depleted with siRNA-mediated knockdown. Two days after siRNA transfection, cells were collected, and proteins were recovered from whole-cell extracts and subjected to western blotting. MED1 and BRCA1 knockdown efficacy was confirmed. siCtrl, MISSION siRNA Universal Negative Control (Sigma Aldrich).

(b) 293T cells were seeded into 6-well plates and transfected the next day with expression

vectors using jetPEI. Cells were harvested 24 h after transfection. Proteins were recovered from whole-cell extracts and subjected to western blotting. MED1 overexpression was confirmed.

(c) Immunoprecipitation was performed with lambda-protein phosphatase, DNase, and RNase in addition to the conditions in Figure 1a. Coprecipitation of MED1 and BRCA1 was inhibited by the addition of all reagents. λ-PPase, lambda protein phosphatase.

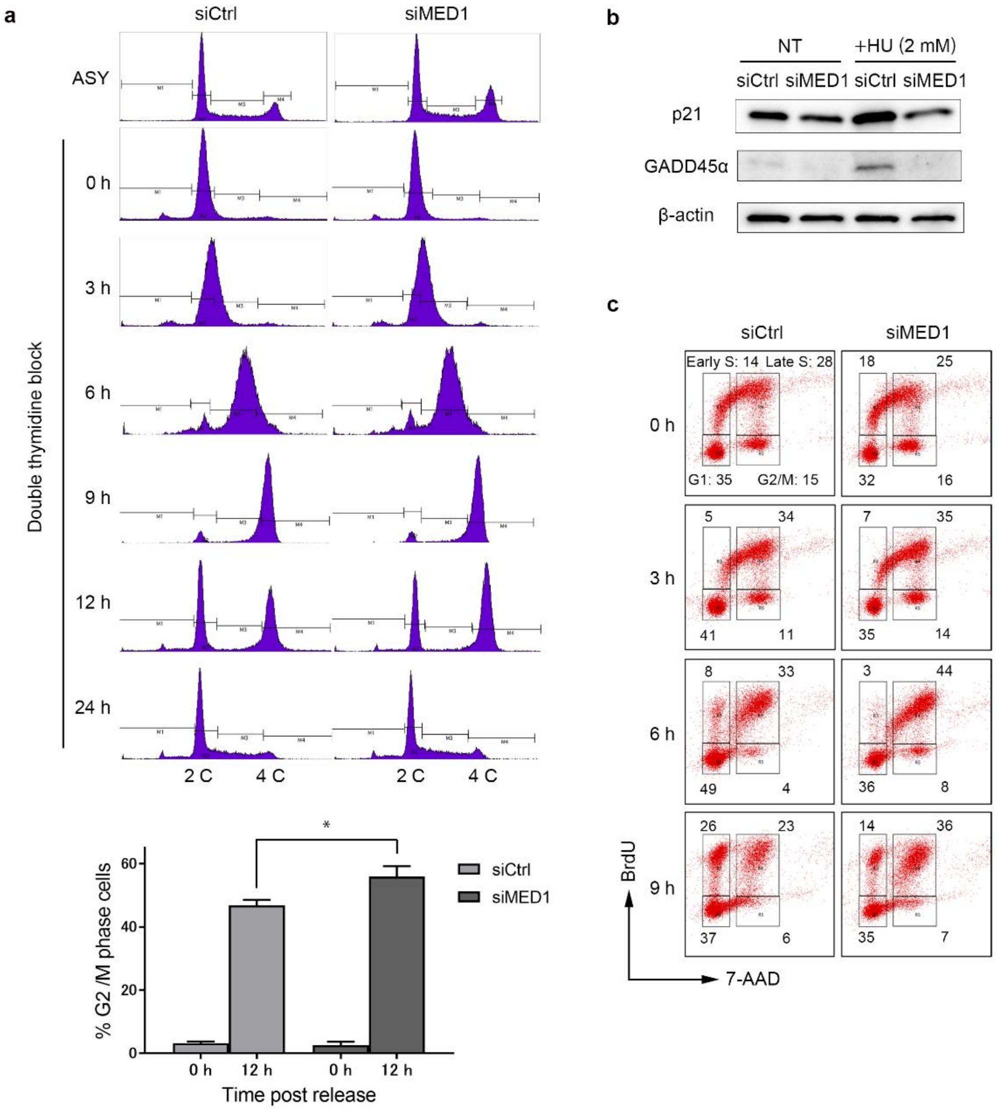

Supplementary Figure S2. Effects of MED1 on the G2/M stage DNA damage checkpoint.

(a) FACS analysis of cell cycle with double thymidine block (DTB) using U2OS and HeLa cells. siRNA transfection was followed by DTB and synchronized to the G1/S phase. G2/M cells were significantly increased in MED1 knockdown cells 12 h after DTB release. Unpaired t-test, *$p < 0.05$.

(b) siRNA knockdown was performed in U2OS cells seeded in 6-well plates. After 2 mM HU treatment for 24 h, cells were collected the next day. Proteins were recovered from whole-cell extracts and subjected to western blotting. p21 and GADD45α expression associated with HU treatment were suppressed in MED1 knockdown cells. siCtrl, MISSION siRNA Universal Negative Control (Sigma Aldrich); HU, Hydroxyurea.

(c) U2OS cells were cultured in 6-well-plates and treated with siRNA knockdown the next day. After 48h, cells were treated with 10 μM BrdU and collected after 0-3-6-9 h. The amount of BrdU was measured by flow cytometry according to the manufacturer's protocol. The results suggest MED1-deficient cells may have delayed S phase progression.

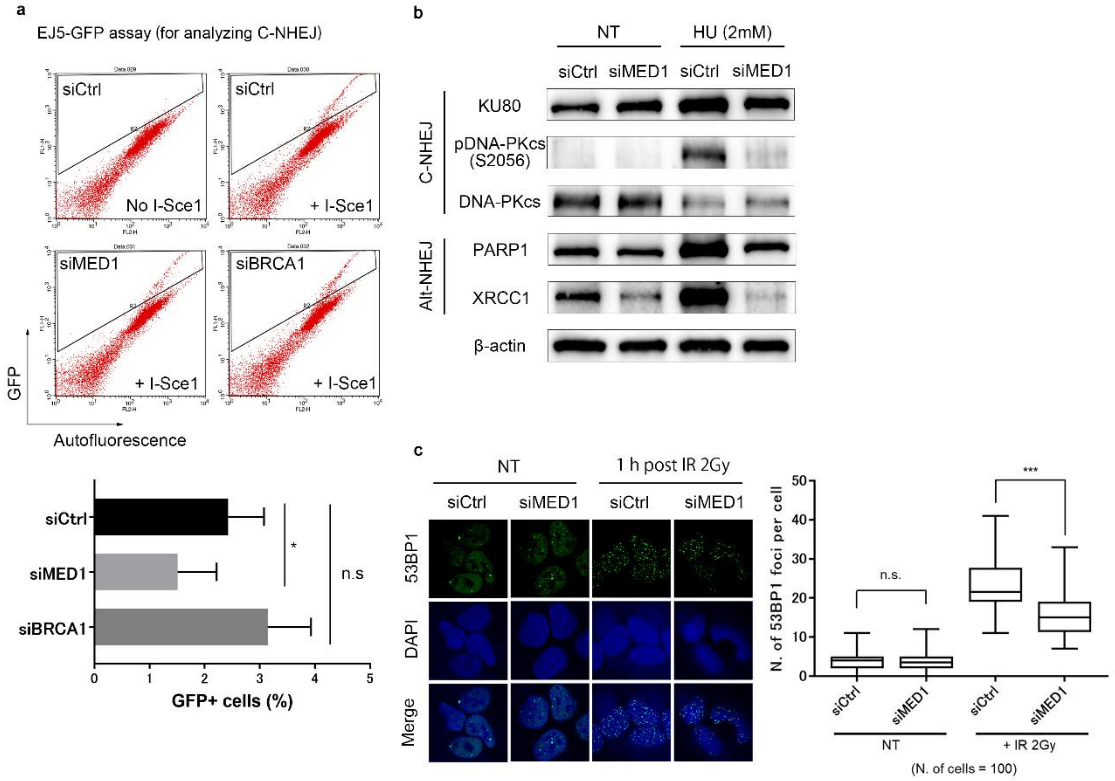

Supplementary Figure S3. Effects of MED1 on the NHEJ pathway

(a) EJ5-GFP U2OS cells were subjected to the same procedure as the DR-GFP assay. MED1 deletion significantly attenuated C-NHEJ activity. Unpaired t-test, * $p < 0.005$.

(b) Western blotting was performed using the protein obtained using the procedure previously described. In MED1-depleted cells, phosphorylation of the C-NHEJ pathway component DNA-PKcs was suppressed, and expression of the Alt-NHEJ pathway proteins PARP1 and XRCC1 were reduced. siCtrl, MISSION siRNA Universal Negative Control (Sigma Aldrich); HU, Hydroxyurea.

(c) siRNA knockdown was performed the day after U2OS cells were seeded. 48h after siRNA

transfection, cells were irradiated with 2 Gy IR and fixed after 1 h. Immunofluorescence was performed as described above. 53BP1 nuclear accumulation was significantly decreased in MED1-depleted cells. siCtrl, MISSION siRNA Universal Negative Control (Sigma Aldrich).

**Supplementary Table 1.**	Used antibodies, plasmids and primer sequences.		
**Primary Antibodies**			
Name	Applicatior	Working concentration	Company
anti-MED1 (A300-793A-M)	WB, IF, IP	1:1000 for western blotting (WB)<br>1:500 for immunofluorescence (IF)<br>1:200 for immunoprecipitation (IP)	Bethyl Laboratories
anti-γH2AX (pSer139) (05-636)	WB, IF	1:1000 for WB<br>1:500 for IF	Merck Millipore
anti-H2AX (#2595)	WB	1:1000 for WB	Cell Signaling Technology
anti-RAD51 (H-92) (sc8349)	IF	1:500 for IF	Santa-Cruz
anti-BRCA1 (#9010)	WB, IF, IP	1:1000 for WB, 1:500 for IF, and 1:200 for IP	Cell Signaling Technology
anti-ATM(D2E2) (#2873)	WB	1:1000 for WB	Cell Signaling Technology
anti-pATM (pSer1981) (EP1890Y)	WB, IF	1:10000 for WB and 1:500 for IF	Gene Tex
anti-ATR (#2790)	WB	1:1000 for WB	Cell Signaling Technology
anti-pATR (pSer428) (#2853)	WB	1:1000 for WB	Cell Signaling Technology
anti-53BP1 (ab36823)	IF	1:500 for IF	Abcam
anti-pChk1 (Ser345)(133D3) (#2348)	WB	1:1000 for WB	Cell Signaling Technology
anti-Chk1(2G1D5) (sc8408)	WB	1:200 for WB	Santa-Cruz
anti-pChk2 (T68) (#2661)	WB	1:1000 for WB	Cell Signaling Technology
anti-Chk2 (DCS-273)	WB	1:1000 for WB	MBL
anti-anti-S9.6 antibody (ENH001)	IF	1:200 for immunofluorescence (IF)	Kerafast
anti-Nucleorin (ab22758)	IF	1:1000 for IF	Abcam
anti-p21 (C-19) (sc397)	WB	1:1000 for WB	Santa-Cruz
anti-GADD45α (#3518)	WB	1:1000 for WB	Cell Signaling Technology
anti-KU80 (#2180)	WB	1:1000 for WB	Cell Signaling Technology
anti-DNA-PKcs (phospho-S2056) (ab124918)	WB	1:5000 for WB	Abcam
anti-DNA-PKcs (ab1832)	WB	1:5000 for WB	Abcam
anti-PARP1 (sc8007)	WB	1:1000 for WB	Santa Cruz
anti-XRCC1 (#2735S)	WB	1:1000 for WB	Cell Signaling Technology
and anti-actin (beta) (A2228h)	WB	1:10000 for WB	Sigma-Aldrich
**Secondary Antibodies**			
Name	Applicatior	Working concentration	Company
anti-rabbit IgG, HRP-linked antibody (#7074)	WB	1:2000 for WB	Cell Signaling Technology
and anti-mouse IgG, HRP-linked antibody (#7076)	WB	1:2000 for WB	Cell Signaling Technology
goat anti-rabbit IgG Alexa Fluor 488 (A27034)	IF	1:500 for IF	Invitrogen
goat anti-mouse IgG Alexa Fluor 568 (A11004)	IF	1:500 for IF	Invitrogen
donkey anti-rabbit IgG Alexa Flour 488 (R37118)	IF	1:500 for IF	Invitrogen
goat anti-rat IgG Alexa Flour 488 (A11006)	IF	1:500 for IF	Invitrogen

Supplementary Table 1.

List of used antibodies in this study
